# Oral health-related quality of life of community-dwellers in the canton of Bern, Switzerland

**DOI:** 10.2340/aos.v84.42707

**Published:** 2025-01-16

**Authors:** Roberta Borg-Bartolo, Andrea Roccuzzo, Christian Tennert, Maria Prasinou, Maurus Jäggi, Pedro Molinero-Mourelle, Michael M. Bornstein, Guglielmo Campus

**Affiliations:** a Department of Restorative, Pediatric and Preventive Dentistry, School of Dental Medicine, University of Bern, Bern, Switzerland; b Graduate School for Health Sciences, University of Bern, Bern, Switzerland; c Department of Periodontology, School of Dental Medicine, University of Bern, Bern, Switzerland; d Unit for Practice-based Research, School of Dental Medicine, University of Bern, Bern, Switzerland; e Department of Reconstructive Dentistry and Gerodontology, School of Dental Medicine, University of Bern, Bern, Switzerland; f Department of Conservative Dentistry and Prosthodontics, Faculty of Odontology, Complutense University of Madrid, Madrid, Spain; g Department of Oral Health & Medicine, University Center for Dental Medicine Basel, University of Basel, Basel, Switzerland; h Department of Cariology, Saveetha Dental College and Hospitals, SIMATS, Chennai, Tamil Nadu, India; i Department of Cariology, Institute of Odontology, Sahlgrenska Akademin, University of Gothenburg, Gothenburg, Sweden

**Keywords:** Oral health-related quality of life, public health, oral health, epidemiology

## Abstract

**Objective:**

The study aims to describe and analyze the oral health-related quality of life (OHRQoL) of persons aged ≥45 years in the canton of Bern, Switzerland.

**Material and Methods:**

Community dwellers were recruited by random sampling from the canton of Bern. Data were collected by a questionnaire (demographic factors, medical history, oral health behavior, dental patient-reported outcomes [dPROs]) and a clinical examination (dental caries, periodontal disease, oral hygiene, mastication). dPROs were evaluated using the OHRQoL-questionnaire Geriatric Oral Health Assessment Index with items related to four domains: functional limitations, pain and discomfort, psychological impact, behavioral impact. χ^2^ tests, Cochrane Armitage trend tests and binary logistic regression were performed with *P* < 0.05 statistical significance.

**Results:**

The highest prevalence (199/44%, *n* [total] = 275 participants) of reported problems was observed in the psychological impact domain. Binary logistic regression shows that participants with rheumatoid arthritis (odds ratio [OR] = 4.86, _95%_ confidence interval [CI] = 1.70–13.87) and chewing deficiencies (OR 28.43, _95%_ CI = 2.11–382.68) had higher odds of having functional limitations, while participants with bleeding gums (OR = 1.69, _95%_ CI = 1.02–2.81) had higher odds of experiencing pain and discomfort. Participants with depression had higher odds of having pain and discomfort (OR = 2.44, _95%_ CI = 1.03–5.81), suffering a behavioral impact (OR 5.89, _95%_ CI = 1.57–22.20) and a low OHRQoL (OR = 2.33, _95%_ CI = 0.09–0.58).

**Conclusions:**

The study shows that poor oral hygiene, high DMFT, chewing deficiency, rheumatoid arthritis, and depression are associated with low OHRQoL.

## Introduction

Dental patient-reported outcomes (dPROs) report directly patients’ perspectives on how they perceive the impact of disease or dental treatment, with the oral health-related quality of life (OHRQoL) concept being the most important dPRO [1]. dPROs are fundamental for evidence-based dentistry, with dental patient-reported outcome measures (dPROMs) measuring what is most important for the patient [2]. The dPRO OHRQoL is used as a measure in different settings, such as clinical practices, to help identify problems and monitor changes and responses to the provided treatments, in survey research as a means of examining trends in oral health, and in clinical studies as an outcome measure with the ultimate goal to improve oral health care [3].

Several oral health conditions have been reported as having an impact on OHRQoL, the most common being tooth loss, edentulism, and poor masticatory performance [4]. Furthermore, oral health is closely related to general health, affecting overall well-being and quality of life [5]. Evidence suggests the presence of a bidirectional association between oral health conditions such as periodontitis and general health conditions, for example, diabetes [6], cardiovascular disease [7, 8] as well as cognitive impairment [9] and depression [10]. The interplay between oral health and general health could be due to physiological factors; periodontitis has been associated with elevated levels of pro-inflammatory cytokines also found in systemic conditions, for example, peripheral artery disease, atherosclerosis, and stroke. People with depression have been found to neglect their oral health, leading to a poor oral health status, as well as side effects associated with medications, such as xerostomia, poor masticatory performance and a poor oral health status [11, 12]. OHRQoL is a multidimensional assessment method that embraces the biopsychosocial model of health into which clinical signs, physical functioning, and emotional and social well-being are incorporated [13]. As the world’s elderly population is increasing [14], and more people are retaining their teeth when aging [15], it is important to know which health factors affect the quality of life, to strive to achieve and maintain a good OHRQoL throughout the life span.

OHRQoL instruments typically consist of multiple-item questionnaires, with the Geriatric Oral Health Assessment Index (GOHAI) [16] being one of the most commonly used [17]. The GOHAI is a 12-item index scored on a Likert scale developed to estimate the degree of psychosocial impact associated with oral diseases in the elderly population. The scale covers physical function, psychological distress and symptoms. When compared to other indices, the GOHAI has been reported to provide useful information when applied to elderly people [18, 19]. Very few studies have used dPROs measuring the OHRQoL concept of adults and elderly persons in Switzerland, especially outside dedicated settings such as hospitals [20], and university clinics [21–23]. Hence, this study aims to describe and analyze the OHRQoL, using the GOHAI, of non-institutionalized persons of at least 45 years of age in the community of the canton of Bern.

## Material and methods

### Study design and target population

Ethics approval for the study was obtained from the Ethical Committee of the Canton of Bern (KEK), Switzerland (Nr. 2020-02760, Nr. 2021-01947), and the study was conducted according to the revised principles of the Helsinki Declaration (2013). Data reporting follows the STROBE guidelines.

The study is a cross-sectional, mono-centric, observational study with participants aged ≥45 years, living in the community in the canton of Bern. Bern is the second largest canton in Switzerland with over one million inhabitants. Proportional allocation, according to the proposed STEP approach guidelines [24], was carried out to sample the individuals from the 10 different regions of the canton of Bern. For the recruitment of participants, the two regions with the smallest proportion of individuals, Obersimmental-Saanen and Frutigen-Niedersimmental, were combined into one (Obersimmental-Frutigen). Details of the applied methodology used were previously reported [25]. Briefly, contact details of participants aged ≥45 years were obtained from the different municipalities of the canton. The potential participants were contacted by mail, where detailed information about the study was provided. Consequently, only participants who agreed to take part in the study and provided written informed consent had an appointment set for the clinical examination, which was carried out at the participants’ place of residence. Participants were excluded if they were under the age of 45, and residing in long-term care facilities or other residential homes. Data collection was carried out between January 2022 and December 2023.

### Sample size calculation

Sample size calculation was performed before the start of the pilot study [25]. As no data was available for Switzerland, an assumption of prevalence of dental caries of 50% was taken, a standard error of 0.05, and a design effect of 2.5 plus an increase of 10%. As the prevalence of active dental caries was found to be lower than assumed, a *post-hoc* power analysis was performed with a prevalence of 15% active dental caries [25] and a prevalence of 9% active dental caries in the present study, a sample size of 275 participants, and a standard error of 0.05. A 0.85 power was achieved.

### Clinical examination and analyzed outcomes

Data were collected using a questionnaire and an oral examination performed at the participants’ homes. The examination was carried out using a plain mirror (Hahnenkratt, Knigsbach, Germany), a WHO ball-ended probe (Asa-Dental, Milan, Italy), and a head torch as light source. Two experienced dentists (AR, RBB), two master dental students (MP, MJ) and four undergraduate dental students carried out the data collection. The examiners were trained and calibrated before the start of the study, with details on the methodology available in a previous publication [25]. Cohen’s kappa scores for intra-rater reliability, and intra-class correlation coefficients (ICC) using the two-way mixed effects model were calculated for inter-rater reliability. The intra-examiner reliability Kappa scores ranged from 80–100% (*p* < 0.05) for dental caries lesion calibration, while 100% (*p* < 0.05) intra-examiner reliability was achieved by all examiners for the dental restoration calibration. The average ICCs for inter-rater reliability were 0.97 (_95%_ confidence interval [CI] = 0.94–0.98, *p* < 0.05) (dental caries, first calibration session), 0.95 (95% CI = 0.90–9.98, *p* < 0.05) (dental caries, second calibration), and 0.98 (_95%_ CI = 0.97–0.99, *p* < 0.05) (dental restoration, first and second calibration).

### Dependent variable

#### OHRQoL

The GOHAI was used as a measure of OHRQoL. The GOHAI is an ordinal variable that is measured on a 5-point Likert scale (1 = always to 5 = never), giving a maximum score of 60. High GOHAI scores indicate a good OHRQoL and vice versa. The questions were categorized into four domains [26]: functional limitations (questions 2, 3, 4), pain and discomfort (questions 5, 8, 12), psychological impact (questions 7, 9, 10, 11), and behavioral impact (questions 1, 6).

### Independent variables

#### Socio-economic factors

The following socio-economic factors were considered: age (45–64 years, 65–74 years, ≥75 years), location (urban/rural with the cut-off taken to be at least 10,000 inhabitants for an area to be considered urban) [27], employment level (in employment/retired), education level (tertiary education/no tertiary education), and civil status (married/not married).

#### General health

The following conditions were considered: cardiovascular disease, gastrointestinal problems, cancer, thyroid disease, diabetes, rheumatoid arthritis, depression, smoking, regular consumption of alcohol.

#### Oral health habits

The following variables were collected: tooth-brushing frequency (at least twice daily, less than twice daily), use of mouthwash (yes/no), use of dental floss and/or interdental brushes (yes/no), frequency of visits to the dentist/dental hygienist (within the last 12 months/over 12 months), sugar consumption (yes/no).

#### Oral health conditions

Oral hygiene: Approximal Plaque Index (API) [28] and modified Papilla Bleeding Index (mPBI) [29]. Good oral hygiene was recorded if <50% of the interproximal spaces had plaque/bleeding, poor oral hygiene was recorded if ≥50% of the interproximal spaces had plaque/bleeding.

Periodontal disease: Periodontal Screening Index (PSI) [30]: The scores range from 0 (healthy periodontal tissue) to 4 (probing pocket depth > 5 mm). Periodontal disease was reported to be present with scores 3–4.

Dental caries (coronal and root caries): ICDAS [31], DMFT. The presence of active dental caries was recorded with scores of ICDAS 4–6 and root ICDAS score of 2. Initial caries lesions were recorded for scores of ICDAS 1–3 and root ICDAS score of 1. DMFT was calculated as follows: D (ICDAS 4–6, root ICDAS 2), F (number of filled teeth), M (number of missing teeth).

Dental prosthesis: removable (full and/or partial dentures, yes/no), fixed (crowns, bridges, yes/no), implants (yes/no).

Masticatory performance: using a two-colored chewing gum mixing test (Hue-Check Gum©, University of Bern, Switzerland). The measures used were variance of hue (VOH) and subjective assessment (SA)[32]; high VOH and SA 1–3 indicated chewing deficiency, low VOH and SA 4–5 indicated no chewing deficiency.

### Statistical analysis

Descriptive statistics were performed with means and standard deviation (SD) to describe continuous variables, and median, inter-quartile range, and frequency (%) and number of participants (*n*) for categorical variables. Binary variables were created for GOHAI sum score and the four domains: functional limitations, pain and discomfort, psychological impact, behavioral impact. For each binary variable, scores 1–3 reported a limitation (1), and scores 4–5 reported no limitation (0). For questions 3, 5, and 7, the scores were inverted to reflect the nature of the question, where, as opposed to the rest of the GOHAI questions, a low score (1) indicates no limitations and a high score (5) indicates a limitation. χ^2^ tests, Fisher tests, and Cochrane Armitage trend tests were performed to assess the crude association between overall OHRQoL and the independent variables. Spearman’s rank correlations were carried out to measure correlations between GOHAI sum score and age, number of missing, filled, and carious teeth, DMFT, and mastication (VOH). A directed acyclic graph (DAG) was constructed using the DAGitty software [33]. DAGs are non-parametric, qualitative graphical tools used to depict causal relations in the epidemiologic assessment of exposure-outcome associations [34]. The DAG shows the outcome variables, that is, the four OHRQoL domains and the exposure variables. The DAG was created to help identify confounding factors which were then included in the regression analysis. Binary logistic regression was performed for the four domains and overall OHRQoL, and odds ratios were reported. A forward logistic regression approach was applied, where logistic regression was run between the outcome variables (the four OHRQoL domains and GOHAI sum score) and independent variables. Where *P <* 0.10 was obtained, the variable was included in the final logistic regression. Effect modifiers were identified, and the model was adjusted accordingly. In the case where a question from the GOHAI was not answered (*n* = 6 questions), a value of 0 was given. There were no missing data for the GOHAI sum score outcome. Statistical analysis was performed using Stata SE18^®^ (StataCorp LLC, College Station, TX, USA) with statistical significance set at *P* < 0.05.

## Results

**Figure 1:**
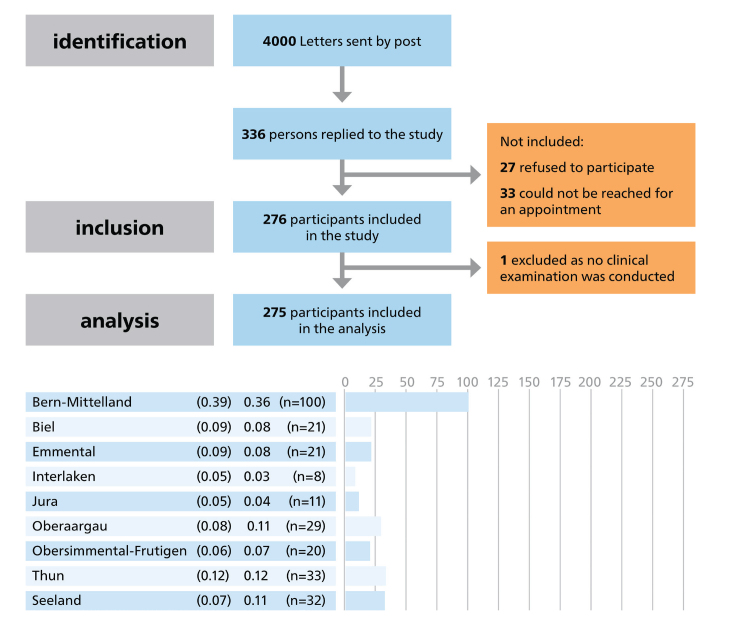
The STROBE Flow Diagram shows the regions from where the participants were recruited, the proportion of the total population aged ≥45 years, the proportion and total of study participants per region.

A total of 275 participants (154/56% males, and 121/44% females), with a mean age of 69.7 (SD 11.6; range 45–99 years) took part in the study. Out of the 4,000 letters sent out, 336 individuals replied to the study invitation. Thirty-three could not be reached for an appointment and 27 refused to participate. In the end, 275 participants filled out the questionnaire and underwent a clinical examination (8% response rate) (Figure 1). The majority lived in rural areas (*n* = 201, 75%) and had a tertiary education (*n* = 146, 53%). A total of 228 (85%) participants reported brushing their teeth twice daily and 196 (80%) visited the dentist every year. It was found that 22 (8%) participants had more than 10 teeth missing. Periodontal disease (i.e. PSI score 3–4) was detected in 108 (40%) participants, and the prevalence of active dental caries (ICDAS 4–6, root ICDAS 2) was 9% (*n* = 25). The median GOHAI was 45 (IQR 30 – 54) with a range of 23 to 60 (Table 1).

**Table 1:** Participants’ characteristics as classified by GOHAI.

Variable	Category	GOHAI ≤ 44	GOHAI ≥ 45	Participants *n* (%)	χ^2^ test	Trend test	
					*p*-value	*z*-value	Exact probability
Sex	Female	51 (33.1)	103 (66.8)	121 (44.0)			
	Male	55 (45.5)	66 (54.5)	154 (56.0)			
				275 (100)	**0.04**	2.09	0.05
Age	45–64 years	31 (39.7)	47 (60.2)	78 (28.0)			
	65–74 years	36 (35.6)	65 (64.4)	101 (36.7)			
	≥ 75 years	39 (40.6)	57 (59.4)	96 (34.9)			
				275 (100)	0.75	−0.17	0.95
Location	Urban	28 (37.8)	46 (62.1)	74 (27)			
	Rural	78 (38.8)	123 (61.2)	201 (73)			
				275 (100)	0.88	−0.15	1.00
Civil status	Married	64 (36.2)	113 (63.8)	177 (65.5)			
	Not married	40 (43.0)	53 (56.9)	93 (34.4)			
				270 (100)	0.27	1.09	0.33
Education level	Tertiary	48 (32.8)	98 (67.1)	146 (53.1)			
	Obligatory-secondary	45 (45.5)	55 (54.5)	101 (36.7)			
	No education/unknown	12 (42.8)	16 (57.1)	28 (10.2)			
				275 (100)	0.12	1.15	0.29
Employment level	In employment	27 (39.7)	41 (60.2)	68 (24.7)			
	Retired	64 (36.3)	112 (63.6)	176 (64.0)			
	Unemployed/unknown	15 (48.4)	16 (51.6)	31 (11.3)			
				275 (100)	0.44	−0.48	0.72
High blood pressure	No	74 (40.2)	110 (59.8)	184 (66.9)			
	Yes	32 (35.2)	59 (64.8)	91 (33.1)			
				275 (100)	0.42	0.81	0.5
Cardiovascular disease	No	86 (36.9)	147 (63.1)	233 (84.7)			
	Yes	20 (47.6)	22 (52.4)	42 (15.3)			
				275 (100)	0.19	−1.31	0.25
Rheumatoid arthritis	No	88 (36.8)	151 (63.2)	239 (86.9)			
	Yes	18 (50)	18 (50)	36 (13.1)			
				275 (100)	0.13	−1.51	0.18
Depression	No	86 (34.8)	161 (65.1)	247 (89.8)			
	Yes	20 (71.4)	8 (28.5)	28 (10.2)			
				275 (100)	**< 0.01**	−3.77	**< 0.01**
Diabetes	No	93 (37.5)	155 (62.5)	248 (90.2)			
	Yes	13 (48.2)	14 (51.9)	27 (9.8)			
				275 (100)	0.28	−1.08	0.40
Tooth brushing frequency	Twice daily or more	88 (38.6)	140 (61.4)	228 (85.7)			
	Once daily or less	13 (34.2)	25 (65.8)	38 (14.3)			
				266 (100)	0.61	−0.51	0.76
Visit to the dentist	In the last 12 months	72 (36.7)	124 (63.3)	196 (79.4)			
	Over 12 months	21 (41.2)	30 (58.9)	51 (20.6)			
				247 (100)	0.56	−0.58	0.67
Visit to dental hygienist	In the last 12 months	69 (34.7)	130 (65.3)	199 (80.6)			
	Over 12 months	24 (50.0)	24 (50.0)	48 (19.4)			
				247 (100)	0.05	−1.97	0.07
Papilla bleeding index (PBI)	<25%	45 ( 40.9)	65 (59.1)	110 (40.7)			
	25%–75%	45 (33.8)	88 (66.2)	133 (49.3)			
	≥ 75%	14 (51.9)	13 (48.2)	27 (10.0)			
				270 (100)	0.17	−1.46	0.18
Approximal plaque index (API)	< 25%	75 (37.0)	128 (63.1)	203 (75.5)			
	25%–75%	22 (40.0)	33 (60.0)	55 (20.4)			
	≥ 75%	7 (63.6)	4 (36.3)	11 (4.0)			
				269 (100)	0.20	−0.19	0.92
Presence of periodontal disease	No	59 (38.3)	95 (61.7)	154 (58.7)			
	Yes	41 (37.9)	67 (62.0)	108 (41.2)			
				262 (100)	0.95	0.06	0.95
Missing teeth	0 missing teeth	21 (30.0)	49 (70.0)	70 (25.5)			
	< 10 missing teeth	72 (39.3)	111 (60.7)	183 (66.5)			
	> 10 missing teeth	13 (59.1)	9 (40.1)	22 (8.5)			
				275 (100)	**0.04**	−2.36	**0.02**
Filled teeth	0–4 filled teeth	29 (41.3)	41 (58.6)	70 (25.8)			
	5–10 filled teeth	32 (33.3)	64 (66.7)	96 (35.4)			
	> 10 filled teeth	43 (41.0)	62 (59.1)	105 (38.7)			
				271 (100)	0.45	−0.09	0.99
Presence of dental caries	No	91 (36.4)	159 (63.6)	250 (91.0)			
	Yes	15 (60.0)	10 (40.0)	25 (9.0)			
				275 (100)	**0.02**	−2.31	**0.04**
DMFT	DMFT < 13	38 (28.4)	96 (71.6)	134 (48.7)			
	DMFT ≥ 13	68 (48.2)	73 (51.8)	141 (51.3)			
				275 (100)	**< 0.01**	−3.38	**< 0.01**
Mastication	Without chewing deficiency	41 (34.8)	77 (65.3)	118 (57.0)			
	With chewing deficiency	34 (38.2)	55 (61.8)	89 (42.9)			
				207 (100)	0.61	0.51	0.60
Removable prosthesis	No	95 (37.3)	160 (62.8)	255 (92.7)			
	Yes	11 (55.0)	9 (45.0)	20 (7.3)			
				275 (100)	0.12	−1.57	0.19
Fixed prosthesis	No	33 (34.0)	64 (65.9)	97 (35.3)			
	Yes	73 (41.0)	105 (58.9)	178 (64.7)			
				275 (100)	0.26	−1.14	0.31
Implants	No	87 (37.5)	145 (62.5)	232 (84.3)			
	Yes	19 (44.2)	24 (55.8)	43 (15.6)			
				275 (100)	0.41	−0.83	0.49

GOHAI: Geriatric Oral Health Assessment Index; DMFT: Decayed, Missing and Filled teeth.

The reliability (Cronbach’s alpha) for GOHAI was 0.80. Out of the four domains, the highest prevalence (199/44%) of reported problems was found in the psychological impact domain, with 102 participants (37%) reporting that they had concerns about their teeth, gums, and/or dental prostheses. Pain and discomfort were experienced by 88 (32%) participants, out of which 66 (24%) reported sensitivity to hot or cold. Forty-seven (17%) participants reported having functional limitations and 23 (8%) reported that their teeth and/or dental prosthesis affected their behavior, particularly limiting food intake and avoiding contact with other people (Table 2).

**Table 2:** Prevalence of problems and limitations in the four OHRQoL domains.

OHRQoL domain	*n* (%)
**Functional limitation**	47 (17.2)
Question 2: trouble biting/chewing hard food	28 (10.0)
Question 3: trouble with swallowing	22 (8.0)
Question 4: trouble with speaking	10 (3.6)
**Pain and discomfort**	88 (32.2)
Question 5: discomfort when eating	24 (8.8)
Question 8: use of medication to relieve pain	20 (7.3)
Question 12: sensitivity of the teeth/gums to hot or cold	66 (24.1)
**Psychological impact**	119 (43.9)
Question 7: Unhappy with appearance of teeth/prosthesis	51 (18.7)
Question 9: worry about the teeth/gum/prosthesis	102 (37.1)
Question 10: uncertain/nervous due to problems with the teeth/gums/dental prosthesis	43 (15.7)
Question 11: uncomfortable eating in front of people due to problems with teeth/dental prosthesis	14 (5.1)
**Behavioral impact**	23 (8.3)
Question 1: limited intake of food due to problems with teeth/dental prosthesis	18 (6.5)
Question 6: avoided contact with other people	9 (3.3)
**GOHAI sum score <45**	106 (38.6)

OHRQoL: oral health-related quality of life; GOHAI: Geriatric Oral Health Assessment Index.

**Figure 2:**
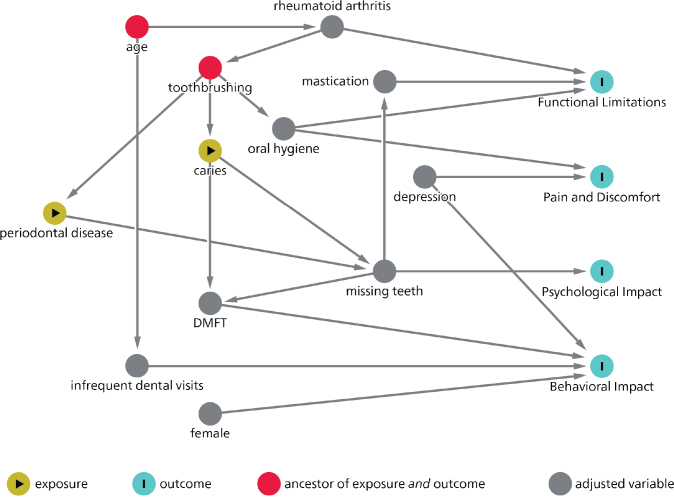
Directed acyclic graph (DAG).

Spearman’s rank correlations between GOHAI sum score and age, number of missing, filled, and carious teeth, DMFT, and mastication (VOH) were not statistically significant. Participants with rheumatoid arthritis (OR = 4.86, _95%_ CI = 1.70–13.87) and those having chewing deficiencies (OR 28.43, _95%_ CI = 2.11–382.68) had higher odds of having functional limitations. Participants with bleeding gums (i.e. high PBI scores) had higher odds of experiencing pain and discomfort (OR = 1.69, _95%_ CI = 1.02–2.81). Suffering from depression was associated with higher odds of having pain and discomfort (OR = 2.44, _95%_ CI = 1.03–5.81), having an impact on behavior (OR 5.89, _95%_ CI = 1.57–22.20) and a low GOHAI sum score (OR = 2.33, _95%_ CI = 1.32–4.34), while participants with a high DMFT had higher odds of experiencing behavioral impact (OR = 1.14, _95%_ CI = 1.02–1.27) and a low GOHAI sum score (OR = 0.94, _95%_ CI = 0.89–0.98) (Table 3) (Figure 2). None of the variables were statistically significant with a psychological impact.

**Table 3:** Binary logistic regression analyses.

Variables	Odds ratio (SE)	*p*-value	95% confidence intervals
**Functional limitation**			
Married	0.57 (0.26)	0.16	0.23–1.28
Rheumatoid arthritis	4.86 (2.60)	**< 0.01**	1.70–13.87
Dental caries	1.18 (0.14)	0.13	0.95–1.50
PBI	2.04 (0.80)	0.07	0.95–4.39
Chewing deficiency	28.43 (37.71)	**0.01**	2.11–382.68
**Pain and discomfort**			
Rheumatoid arthritis	1.45 (0.60)	0.37	0.65–3.25
Depression	2.44 (1.08)	**0.04**	1.03–5.81
Brushing twice daily or more	2.32 (1.06)	0.07	0.95–5.69
PBI	1.69 (0.44)	**0.04**	1.02–2.81
**Psychological impact**			
GI problems	2.00 (0.75)	0.06	0.97–4.15
Depression	2.29 (1.08)	0.08	0.91–5.79
Having a fixed dental prosthesis	1.64 (0.46)	0.08	0.94–2.84
PBI	1.58 (0.42)	0.09	0.93–2.69
Missing teeth	1.07 (0.03)	0.05	1.00–1.14
**Behavioral impact**			
Male	0.14 (0.09)	**<0.01**	0.04–0.54
Depression	5.89 (4.00)	**<0.01**	1.57–22.20
Use dental floss	0.34 (0.23)	0.11	0.09–1.27
Visit to the dentist > 12 months	4.07 (2.63)	**0.03**	1.14–14.47
Visit to the hygienist > 12 months	2.75 (1.72)	0.11	0.81–9.34
PBI	1.94 (0.75)	0.09	0.90–4.15
DMFT	1.14 (0.06)	**0.02**	1.02–1.27
**GOHAI sum score**			
Male	2.40 (0.73)	**<0.01**	1.32–4.35
GI problems	0.63 (0.26)	0.26	0.28–1.41
Rheumatoid arthritis	0.67 (0.30)	0.38	0.27–1.63
Depression	0.23 (0.11)	**<0.01**	0.09–0.58
Visit to the hygienist > 12 months	0.64 (0.24)	0.25	0.31–1.35
PBI	0.76 (0.21)	0.32	0.44–1.30
Caries present	0.45 (0.23)	0.12	0.16–1.24
DMFT	0.94 (0.03)	**0.02**	0.89–0.98

SE: standard error; PBI: Papilla bleeding index; DMFT: Decayed Missing and Filled Teeth; GI: Gastrointestinal problems; GOHAI: Geriatric Oral Health Assessment Index.

## Discussion

The present study identified several factors (depression and rheumatoid arthritis, infrequent dental visits, poor oral hygiene, chewing deficiency, and a high DMFT) that contribute to a low OHRQoL, with almost half of the participants reporting psychological impact, and a third presenting with pain and discomfort.

The association between oral health factors and OHRQoL is well-documented [35]. More specifically, a high number of teeth is associated with a good OHRQoL [36, 37], while poor masticatory performance has a negative association with OHRQoL [38, 39]. In this respect, our findings corroborate previous investigations. When considering the potential link between dental caries, poor oral hygiene, bleeding gums, and periodontal disease with OHRQoL [4], at the time being, there is a lack of consensus [4]. Dental caries and periodontal disease, however, do not always cause pain and may not be located in the esthetic zone, thus not influencing directly the self-reported OHRQoL. However, if left untreated, these conditions could eventually lead to pain and tooth loss, could affect masticatory performance, thus influencing food choices and nutrition [40], and could lead to sleep problems as well as poor social interactions, thus ultimately impacting OHRQoL [4]. In the present investigation, dental caries and periodontal disease were not found to be significantly associated with OHRQoL, although DMFT was. The D (decayed teeth) and M (missing teeth) components of the DMFT-index were statistically significant in the χ^2^ testing and trend tests performed, as well as in the unadjusted logistic regression model with the GOHAI sum score. However, when an adjusted model was implemented, such statistical difference was no longer detected. This could be a possible explanation as to why overall DMFT was found to have a statistically significant association with OHRQoL, while the individual components were not.

Numerous studies have reported an association between general health and OHRQoL. Conditions such as Parkinson’s disease [41], frailty [42], dental anxiety [43], and depression have all been found to have a negative impact on OHRQoL. In the present study, depression was found to be associated with three out of the four OHRQoL domains as well as with the GOHAI sum score. Several research findings have reported an association between depression and low OHRQoL [10, 44, 45]. Specifically, a bidirectional association was found between depression and poor oral health: people with poor oral health and no depressive symptoms were more likely to report symptoms of depression after several years of follow-up, while people with depression and good oral health were more likely to report poor oral health compared with people without depressive symptoms [46]. General aspects of health could potentially directly influence oral health, which in turn influences the quality of life related to oral health. Studies have shown that people with depression are more likely to neglect their oral hygiene, which in turn leads to problems like halitosis, periodontal disease, dental caries and possible tooth loss [46]. Antidepressants may cause xerostomia and trouble swallowing, negatively impacting the quality of life [47]. Consequently, these limitations may lead to limited social interactions and poorer mental health [48]. Such interactions demonstrate the interconnection between the biological, psychological and socio-environmental factors. In Switzerland, as in most countries worldwide, minor depression is widespread among adults [49]. Therefore, such findings should not be underestimated.

The second condition significantly associated with low OHRQoL in this survey was rheumatoid arthritis. People with rheumatoid arthritis have difficulty maintaining good oral hygiene [50], and present with a high incidence of periodontal disease, TMJ dysfunction, and salivary gland dysfunction [51]. It is consequently of paramount importance that the whole dental team recognizes potential complications that can arise due to rheumatoid arthritis or its treatment, to successfully manage the patient with early intervention to prevent further decline in quality of life [51].

It would have been interesting to compare the findings with other studies conducted at a national level. However, to the best of the authors’ knowledge, only one study has used dPROs to measure the OHRQoL in a community-dwelling elderly population, who were care-dependent, using the Oral Health Impact Profile [52]. Thus, a direct comparison between the two studies is not possible. When comparing the results obtained with those from neighboring countries (France and Germany) with similar study populations [40, 53], the present study reported a lower GOHAI sum score. In the present study, the highest prevalence of reported problems was in the psychological impact domain, with most participants scoring low due to nervousness and concern about their dental status and unhappiness with their appearance. Evidence suggests that dental anxiety is a psychological determinant that has an impact on OHRQoL [54]. People of different nationalities and cultural backgrounds have different perceptions of oral health [55], with studies reporting differences in the OHRQoL among immigrants and ethnic groups [56], as well as among people of different religions [57]. This difference applies especially for health, which is dynamic and depends on the environment one lives in [58]. In Switzerland, oral health care differs from that of France and Germany. While in Switzerland, dental care is primarily self-paid [59], France and Germany have a social health insurance system, that provides extensive coverage of dental health care [60]. Such variation could potentially explain the differences noted in the OHRQoL in the present study when compared to neighboring countries.

From a methodological point of view, this study presents several strengths, including the random sampling of the participants and the good representation of all the 10 regions of the canton of Bern (urban and rural) as well as the high power of the study. Moreover, the addition of clinical data following the performed clinical examinations to the self-reported data has provided a more comprehensive view of the oral health situation. However, the study is not free from limitations: firstly, the overall participants’ response rate was low, even though comparable to similar studies conducted in Switzerland [52] One reason for this could be the COVID-19 pandemic, where participants, especially elderly persons, might have been reluctant to take part. Other reasons could be poor general health, which would limit the ability to participate, and poor oral health, which would result in an unwillingness to participate in such research initiatives. A lack of trust in research staff and a lack of perceived benefit were reported as reasons for older people not participating in research activities [61]. Secondly, although every effort was made to include people from different backgrounds, by randomly selecting the participants from the contact lists of the municipalities, a potential participation bias cannot be excluded since the majority of the participants were highly educated, leading to people from lower education backgrounds being under-represented. Thus, the study might portray a situation better than it is. However, it is important to note that even though participants had a good education level with good oral health behavior, the GOHAI sum score was low, with problems and limitations reported in the OHRQoL domains.

The findings of the study highlight the need for more research, preferably in the form of longitudinal studies to enable causal interpretation of the results, on the OHRQoL of the adult and elderly population living in their own homes. Further studies should make every possible effort to minimize bias associated with low response rates and participation to ensure the representation of the whole population. In clinical practice or for research purposes, using dPROs to measure the OHRQoL is a straightforward procedure that is minimally time-consuming, which could help detect problems and improve patient management. Given that general health, directly or indirectly impacts OHRQoL, such measurements should be carried out more frequently, not only in the dental field but also by other healthcare professions. Oral health has been historically isolated from medicine, with oral health and general health being treated separately [62]. This can be seen even nowadays, for example, in how health care systems are set up, with dental care very rarely being integrated in primary care [63]. Given the well-established association that general health has with oral health, the involvement of healthcare professionals from various specialties including doctors, nurses, dieticians, speech therapists and caregivers is essential to improve oral health. Furthermore, measuring the OHRQoL on a population level, by including this measurement in national oral health surveys, is recommended. Among other indicators, measuring OHRQoL on a macro level is useful as a factor in evaluating access to dental care. Access to dental care is an issue prevalent in both developed and developing countries, albeit for different reasons, thus measuring OHRQoL is useful in measuring the impact of oral health disparities on oral health and quality of life [64].

## Conclusion

Within its limitations, the outcomes of the present study highlight that rheumatoid arthritis and depression, as well as, poor oral hygiene, a high DMFT, and chewing deficiency, were all contributors to a low OHRQoL in middle-aged and elderly participants living in the canton of Bern. Such findings underline the importance of maintaining good oral health throughout the life course. Furthermore, given the association of general medical conditions with OHRQoL, the dental team as well as other healthcare professionals must be aware of the impact that general health has on oral health and subsequently, on the quality of life.

## Data Availability

Data will be made available by the corresponding author upon reasonable request.
